# Effect of an Education Programme on Cardiovascular Health Index among Patients with Myocardial Infarction: A Preliminary Study

**DOI:** 10.21315/mjms2018.25.2.11

**Published:** 2018-04-27

**Authors:** Norazlin Ab Manap, Siti Khuzaimah Ahmad Sharoni, Padma A Rahman, Hayati Adilin Mohd Abdul Majid

**Affiliations:** 1 Kolej Sains Kesihatan Bersekutu Sungai Buloh, Jalan Hospital, 47000 Sungai Buloh, Selangor, Malaysia; 2 Centre for Nursing Studies, Faculty of Health Sciences, Universiti Teknologi MARA Selangor, Puncak Alam Campus, 42300 Puncak Alam, Selangor, Malaysia; 3 Centre for Occupational Therapy, Faculty of Health Sciences, Universiti Teknologi MARA Selangor, Puncak Alam Campus, 42300 Puncak Alam, Selangor, Malaysia; 4 Centre of Food Service, Faculty of Hotel & Tourism Management, Universiti Teknologi MARA, UiTM Cawangan Terengganu, Kuala Terengganu Campus, 21080 Chendering, Terengganu, Malaysia

**Keywords:** education, cardiovascular diseases, prevention and control, myocardial infarction

## Abstract

**Introduction:**

Health education is an essential part of controlling the risk of myocardial infarction (MI). This study evaluates the effects of one-on-one education programmes on the cardiovascular health index among patients with MI.

**Methods:**

A quasi-experimental study was conducted in Kuala Lumpur Hospital, Malaysia. Data were collected from November 2014 to January 2015 with a total of 58 respondents who met the inclusion criteria. The respondents received a 20-min one-on-one education programme regarding coronary heart disease, treatment and prevention, and healthy lifestyle. A questionnaire comprising demographic data was administered and the cardiovascular health index was measured before and after four weeks of the education programme. Data were analysed with descriptive and inferential statistics.

**Results:**

There were statistically significant decreases in the score of anxiety, stress, depression, body mass index, and smoking status (*P* < 0.001) between pre-test and post-test.

**Conclusion:**

The findings suggest that the one-on-one education programme could improve the cardiovascular health index of patients with MI. Furthermore, nurses need to develop and implement a standard education structure programme for patients with MI to improve health outcomes.

## Introduction

Myocardial infarction (MI) is a leading cause of morbidity and mortality. Annually, more than three million people are estimated to have acute MI, and more than four million have MI (1). In Malaysia, ischaemic heart disease accounted for 12.9% of medically certified deaths in 2008 (2).

The definition of cardiovascular health index is based on seven health factors and behaviours: smoking, body mass index (BMI), diet, physical activity, blood pressure, and glucose and cholesterol levels (3). The measurement of cardiovascular health index items such as smoking, BMI, blood pressure, and psychological factors may prevent the recurrence of the disease (4). Quitting smoking improves health outcomes among patients post-MI and increases their survival rate (5). Systolic blood pressure (SBP) and diastolic blood pressure (DBP) are predictors and markers of the risk of MI and cardiovascular events (6, 7). Furthermore, obesity and overweight are associated with the risk of acute MI, followed by mortality (8).

Besides the physical and clinical effects, MI may have a large impact on psychological dimensions. Patients with MI may show negative psychological reactions such as depression, anxiety, and stress (9). These emotional reactions were found to be associated with an increase in mortality risk (10, 11). Anxiety and depression were the major reasons patients did not comply with rehabilitation programmes (12).

The effectiveness of health education programmes has become an important issue in managing patients with MI (13, 14). Many studies have shown that health education can improve cardiovascular health index items through the promotion of health-related behaviours, increase compliance rate and improve quality of life (4, 15–18). In cardiac teaching programmes, patients will be taught about the diseases, risk factors, compliance with medication, diet regime, regular exercises, and stress reduction (19). The implementation is when the teaching plans are put into practice and evaluated to determine whether the patient is in compliance and whether a change in their behaviour has occurred (20).

However, few studies have examined health education programmes specifically for patients with MI in local settings. Additional research addressing the relationship between a cardiovascular health education programme and the outcomes in this setting is crucial. Therefore, this study proposes to contribute new data pertaining to patients with MI in Malaysia. Thus, this study implements and evaluates the effects of a one-on-one education programme on cardiovascular health index among patients with MI. The results will help enhance patient care quality by providing them with sufficient information to help them change their health status.

## Methods

This is a one-group, pre- and post-test, quasi-experimental study design. The study was conducted in the coronary care unit (CCU) and coronary rehabilitation programme (CRP) clinic, Kuala Lumpur Hospital. The inclusion criteria were patient diagnosed with MI for the first time, aged between 40 and 70 years old, non-medical staff or having a medical background and able to read and write in Malay. Patients who planned to be transferred to other hospitals for cardiac catheterisation or coronary artery bypass graft were excluded from this study.

A probability systematic sampling technique was used where respondents were identified from the registration book. The respondents were selected based on the odd numbers in the name list. The sample size was calculated based on a previous study (4) using a formula from Lemeshow (21). The average score at pre-test and post-test for anxiety in the study was 17.31 (SD = 11.03) and 11.95 (SD = 7.6), respectively (4). The hypothesis of no difference is to be tested at the (α) 0.05 level of significance and power (1−β) = 0.80 (2-tailed). Approximately 66 respondents were required to participate in this study; however, an additional number of respondents were required to retain 10% of potential attrition. Therefore, 72 respondents were required for this study.

### Instruments

The questionnaire was divided into Parts A, B, and C. Part A comprises demographic questions such as age, gender, race, education level, occupation, and total income per month. Demographic data were collected at pre-test, before the education programme was conducted, as baseline information.

Variables of the cardiovascular health index measured at pre-test and post-test consisted of Part B (psychological elements) and Part C (clinical data). In Part B, the validated Malay version of the Depression Anxiety Stress scale (DASS) 21 was adopted to measure the three subscales of psychological aspects: depression, anxiety, and stress (22). The original version of the DASS 21 was constructed and validated by Lovibond in 1995 (23, 24). This instrument contains 21 items (seven items measuring each subscale), measured with a Likert scale (0 = never, 1 = sometimes, 2 = often, 3 = almost always). The cut-off of this instrument was divided into five categories: normal, mild, moderate, severe, and extremely severe (22). Higher scores on each scale indicate that the level of depression, anxiety and stress of respondents increased (25). It was reported that the internal consistency of the Malay-DASS 21 was good, and the results show that the values of depression, anxiety and stress were 0.84, 0.74 and 0.79, respectively (22). The overall internal consistency result of the Malay-DASS 21 in this study was 0.74.

Part C comprised four items concerning the clinical data of the cardiovascular health index: smoking status, measurement of SBP, DBP and BMI. The respondents were asked about their current smoking status for the past month. Smokers were identified if the respondents smoked at least one cigarette within one month and respondents who never smoke at all or previously smoked (more than one month) were identified as non-smokers (26). The measurement of SBP, DBP and BMI were determined by the patient’s record between 24 h and 48 h after the respondents were admitted to the CCU.

In practice, patients in the CCU were on cardiac monitoring, and a staff nurse in charge recorded the vital signs (SBP and DBP measurement) in the patient’s observation chart. The average values of the latest measurements, first and second systolic and diastolic values, were reported as the SBP and DBP value for each respondent. A SBP and DBP value of < 120 mmHg and < 80 mmHg is considered normal (27). BMI was calculated as (weight [kg]) divided to (height^2^ [metres]^2^). The BMI was classified as: < 18.5 kg/m^2^ = underweight; 18.5–22.9 kg/m^2^ = normal; 23–27.4 kg/m^2^ = overweight; > 27.5 kg/ m^2^ = obese (28).

The education material consisted of a leaflet on the cardiovascular health index. The leaflet titled ‘Penyakit Jantung Koronari’ (coronary heart disease) was adopted with permission from the cardiology unit, University Malaya Medical Centre (UMMC). The leaflet has been used for health education programmes for patients with MI in UMMC since 2009. The content of the leaflet covers the following information: introduction, how and when MI happens, signs and symptoms of MI, risk factors, early management at home, medical treatment and prevention, and lifestyle adjustment (smoking cessation, stress management, medication, exercise and weight reduction, diet, and follow-up). An expert panel comprising one cardiologist, one lecturer, two cardiac care nurses and one patient with MI, reviewed the face and content validity of the leaflet. This process was done to justify that the leaflet was appropriate and practical use for the respondents in the CCU and the CRP Clinic, Kuala Lumpur Hospital.

### Ethical Consideration

Ethical approval to conduct the study was obtained from the Research Ethic Committee, Faculty of Health Sciences, Universiti Teknologi MARA (UiTM), reference no: 600-FSK (PT.5/2). Since the data were collected from a public hospital, the study was registered with the National Medical Research Register (NMRR), National Institute of Health Malaysia and Medical Research and Ethic Committee (MREC) (MREC ID: NMRR-14-965-22024). All the respondents were informed about the purpose of the study. Consent forms were signed, and participation was voluntary with respondents having the right to withdraw from the study at any time. To ensure confidentiality, the data were kept in a sealed envelope, each respondent was coded with a separate number, and a letter ‘A’ for the pre-test and letter ‘B’ for the post-test.

### Data Collection Process

The data collection process was conducted from November 2014 until January 2015. Respondents were selected based on their diagnosis during hospitalisation and met the criteria of the study. At day two (after 24–48 hours of admission), where the respondents were considered medically stable, questionnaires were distributed to the respondents. Clinical data measurements (Part C: smoking status, measurement of SBP, DBP and BMI) were taken after all the respondents completed the questionnaire in Part A (demographic data) and Part B (depression, anxiety, and stress). Each respondent took approximately 20 min to complete the questionnaire. They were then informed that the post-test would be held after four weeks during their first follow-up visit at the CRP clinic. Data collection was conducted by a trained enumerator (registered nurse) from pre-test to post-test (week 4).

On day 3, the respondent received approximately 20 min of one-on-one education programme aided with a leaflet. The main researcher (experienced Cardiac Care Nurse) delivered the session in the respondent’s room (CCU unit). At the end of the session, the respondents were encouraged to ask for further clarification (question and answer), and were advised to keep the leaflet for their reference.

In week 4, during their first follow-up visit at the CRP clinic, the respondents were evaluated (post-test) for the outcome measures (cardiovascular health index). The respondents were asked to complete the questionnaire in Parts B (psychological elements) and C (smoking status). The information of SBP, DBP and BMI at week 4, were determined from the patient’s record by the enumerator.

### Statistical Analysis

Data were analysed with the Statistical Package for the Social Sciences (SPSS) version 20.0. Descriptive statistics (mean and standard deviation [SD] or frequency [*n*] and percentage [%]) were used to analyse the demographic and cardiovascular health index data. Normality of the continuous data was assessed with skewness and kurtosis, indicating that the majority of the variables had a normal distribution as the values were between −2 and +2. The effects of a one-on-one education programme on cardiovascular health index among patients with MI between pre-test and post-test were analysed with a paired *t*-test for continuous data and McNemar’s test for categorical data.

## Results

**Figure 1:**
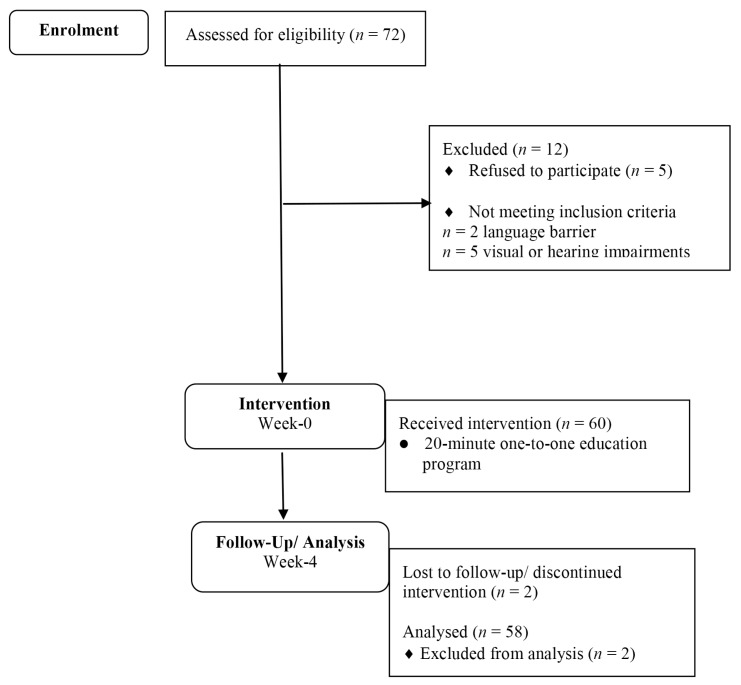
Flow Diagram of enrolment, intervention, follow up and analysis of the study. The figure was modified from CONSORT 2010 Flow Diagram

A total of 72 questionnaires were distributed to the respondents. However, 14 of them were excluded for this analysis for the following reasons: refused (5), language barrier (2), visual or hearing impairment (5) and not able to complete the second measurement (post-test) due to loss of follow-up (2). Therefore, only 58 participants responded to the pre-test and post-test, providing a response rate of 81% (Figure 1).

### Demographic Data of Respondents

As can be seen in Table 1, the average age and income were 55 years and RM 2763.8 (SD = 691.5), respectively. Most of the respondents were Indian (44.8%), men (79.3%), attended secondary school (60.3%) and worked in the non-professional sector (63.8%).

### Distribution of the Cardiovascular Health Index Data (Pre-test)

As can be seen in Table 2, the majority of respondents recorded high to extremely high levels of anxiety, stress, and depression. All respondents showed the normal classification of SBP. For the DBP, 10.3% of the respondents were classified as having an abnormal condition (DBP: > 80 mmHg). The BMI showed that more than 55% of the respondents were overweight with the prevalence of obesity at higher than 10%. Most of the respondents were smokers (87.9%).

### The Differences in Cardiovascular Health Index Measurement between Pre-test and Post-Test

Table 3 presents the differences in the cardiovascular health index measurement before and after the education programme. The analysis shows that at pre-test, the average score of anxiety (M = 18.26, SD = 2.70), stress (M = 15.55, SD = 2.97), depression (M = 18.17, SD = 3.06) and BMI (M = 26.41, SD = 2.08) were statistically decreased at the post-test (anxiety: M = 10.34 [SD = 3.49), *P* < 0.001; stress: M = 9.14 [SD = 3.70], *P* < 0.001; depression: M = 11.28 [SD = 4.13], *P* < 0.001; BMI: M = 25.79 [SD = 2.02], *P* < 0.001). The number of smokers was reduced by more than 50% after the education programme (*P* < 0.001).

## Discussion

### Demographic Data of Respondents

Demographic data are important variables as an individual’s socio-economic background may affect the survival and the recurrence in patients post-MI (29). The results showed that the majority of the patients were men and found an average age of 55 years. Other studies found that the majority of their samples were men and the mean age was 55 years (14, 16). Indian patients were the most common respondents. Lu and Nordin’s study found that Chinese and Indian people were at lower risk of acute coronary syndrome compared to Malays (30). In line with other studies, the majority had secondary education, were working in non-professional jobs (31) and had average income (4). This may be due to the majority of respondents being aged 55 years and working as non-professionals.

### Distribution of the Cardiovascular Health Index Data

The majority of the respondents were smokers and had extremely high levels of depression, anxiety, and stress. Other studies stated that patients experienced depression post-MI (15, 32–34) and anxiety (4). It has been reported that most patients with MI were smokers (16, 35), and smoking is one of the factors that contributed to MI. This study showed that more than half of the respondents were overweight and a few were obese. Previous studies also found that most of their respondents were overweight and obese (4, 15, 16). The result from this study was similar to that of previous studies, (4) which found that most of the respondents have normal blood pressure (SBP and DBP) in the results of the pre-test. This may be due to the data collection period where the patients’ cardiovascular health index was measured 24–48 h after admission. During this period, patients’ health condition usually improved.

### The Differences in Cardiovascular Health Index Measurement between Pre-Test and Post-Test

The primary objective of this study was to evaluate the changes in the cardiovascular health index measurement after implementing the education programme. There was an improvement in the cardiovascular health index scores (depression, anxiety, stress, BMI, and smoking status) from pre-test to post-test.

#### Psychological elements (depression, anxiety, and stress)

Psychological factors are not commonly the main focus in the management of patients with MI, because healthcare providers are more concerned about the damage to the patient’s heart, without knowing that the psychological factors may worsen the prognosis of the disease (36). This study revealed that the levels of depression, anxiety and stress were extremely severe before the education programme, and were lower at the post-test. Other studies also showed that cardiac health education could reduce anxiety and depression levels and help in the rehabilitation phase of patients with MI (4, 37). This type of programme helps patients cope with their current psychological factors for post-MI (38). Education programmes can also decrease anxiety for patients of pre-coronary artery bypass surgery (39).

Patients with MI find it very difficult to adjust to their new life. For this reason, psychological support is crucial and should be focused on in the management of MI. Patients who received psychological support have the highest quality of life and reduced risk of recurring infarction (40). Lifestyle changes towards the positive environment and stress management are necessary. Therefore, cardiac nurses need to implement a special health education programme for patients with MI. During the education programme, nurses need to identify the patients’ feelings and psychological status. Nurses can involve the patients’ families in the programme as part of the family support.

#### Clinical data (BMI and smoking status)

The data showed a reduction in BMI and smoking between pre-test and post-test of the education programme. Similarly, other studies have reported an improvement in BMI (4, 15, 41) after implementation of health education programmes among patients with cardiac problems.

Hence, patients with MI should be provided with sufficient knowledge on how to manage their body weight. Patients need to be educated about the benefits and consequences of weight loss. This includes a change of lifestyles such as reducing dietary intake that is high in fat, calories, and sodium. Involvement in physical activity and the measurement of BMI among patients with MI should be compulsory. Monitoring body weight and BMI consistently is crucial to prevent further heart-related health complication. Weight is directly associated with blood pressure and there is a proven relationship between one risk factor and the other (42). Controlling the associated factors in cardiovascular disease is essential to reduce the chances of contracting these diseases and/or it recurring.

In this study, a number of smokers showed improvement after the education programme. Similarly, other studies have found that health education helped to increase quitting smoking among patients with MI (4). Cardiac nurses can play an active role in helping smokers to quit through well-organised smoking cessation programmes (43). Therefore, there is a need to develop an integrated event about quitting smoking into relevant health programmes. For example, referring them to smoking quitting clinics is essential. Support groups on smoking cessation can be developed so that they can share their tips on reducing the habits of smoking.

#### Study limitations

First, as there was no control group in this quasi-experimental study, there is a high risk of potential biases. A control group was not available to determine the effectiveness of study between groups. Moreover, the duration of four weeks for the programme was inadequate to assess the sustainability of the programme in terms of the outcome measures. This study was conducted at one hospital, which limits the ability to generalise the findings to other populations. Other cardiovascular health index components such as diet, physical activity, and blood investigations (plasma glucose and total cholesterol) were not measured in this study. This study involved a self-report questionnaire and variables of SBP, DBP and BMI were taken solely from the patient’s record.

Future research on health education among patients with MI can be conducted using other vigorous research designs. The evaluation of long-term effects (more than four weeks follow-up) is warranted to determine the sustainability of the programme in improving the cardiovascular health index. For instance, randomised control trials can reduce any potential bias in terms of research methodology. Other designs, such as mixed method or a qualitative study based on theory-behavioural education may provide additional knowledge from different points of view and outcomes.

## Conclusion

The preliminary study results support that the patients who received the one-on-one education programme showed an improvement in their level of depression, anxiety, stress, BMI, and smoking status. In view of professional development, all cardiac nurses should prepare and equip themselves with effective teaching and learning strategies. It has been reported that nurses still lack knowledge on the foundation of effective patient teaching and education (44). This highlights the importance of health education programme for patients with MI to improve their health. Nurses may take this opportunity to develop their knowledge and skill in health education to better meet patients’ healthcare needs. These strategies can enhance the nurses’ competency levels while delivering education programmes to patients with MI.

It is highly recommended to have cardiac nurse specialists to transfer their knowledge through such health education programmes to patients with MI before discharging them. This will improve patients’ knowledge, reduce psychological uncertainty, and may help them to cope with their new lifestyle. The hospital administration needs to develop a leaflet or other source of information regarding MI for the patients in Kuala Lumpur Hospital. A series of workshops on MI topics for patients with MI are also required to enhance the patients’ awareness, prevent recurrence of sudden heart attack, and improve disease management.

## Figures and Tables

**Table 1:** Distribution of the demographic data (*n* = 58)

Continuous variable		*n*	%
Age (Mean, SD)		55.0 (10.3)	
Income (RM)		2763.8 (691.5)	
Gender	Male	46	79.3
	Female	12	20.7
Race	Malay	24	41.4
	Chinese	8	13.8
	Indian	26	44.8
Education level	Primary	11	19.0
	Secondary	35	60.3
	Tertiary	12	20.7
Occupation	Not working	12	20.7
	Non-professional	37	63.8
	Professional	9	15.5

**Table 2:** Distribution of the cardiovascular health index data (pre-test; *n* = 58)

Variables		*n*	%
Anxiety	Moderate	N/A	
	Severe	N/A	
	Extremely severe	58	100
Stress	Mild	3	5.2
	Moderate	8	13.8
	Severe	10	17.2
	Extremely Severe	37	63.8
Depression	Severe	3	5.2
	Extremely Severe	55	94.8
SBP	Normal	58	100
	Abnormal	N/A	
DBP	Normal	52	89.7
	Abnormal	6	10.3
BMI	Underweight	N/A	
	Normal	17	29.3
	Overweight	33	56.9
	Obese	8	13.8
Smoking status	Yes	51	87.9
	No	7	12.1

**Table 3:** The differences of cardiovascular health index measurements between pre-test and post-test (*n* = 58)

Variables	Pre-test	Post-test	Test Statistics	*P*-Value
	Mean (SD)			
Anxiety score^a^	18.26 (2.70)	10.34 (3.49)	13.300	*P* < 0.001
Stress score^a^	15.55 (2.97)	9.14 (3.70)	11.059	*P* < 0.001
Depression score^a^	18.17 (3.06)	11.28 (4.13)	10.593	*P* < 0.001
SBP^a^	121.21 (9.66)	118.45 (10.56)	2.822	0.007
DBP^a^	81.55 (12.40)	79.83 (10.68)	1.693	0.096
BMI^a^	26.41 (2.08)	25.79 (2.02)	4.700	*P* < 0.001
Smoking status^b^ (yes, *n*%)	51 (87.9)	18 (31.0)	31.030	*P* < 0.001

Note:

a Paired *t*-test was used to compare the means between pre-test and post-test for continuous data;

b McNemar’s was used to compare the proportion between pre-test and post-test for categorical data.
